# Future Perspectives on GLP-1 Receptor Agonists and GLP-1/glucagon Receptor Co-agonists in the Treatment of NAFLD

**DOI:** 10.3389/fendo.2018.00649

**Published:** 2018-11-06

**Authors:** Marta Seghieri, Alexander S. Christensen, Andreas Andersen, Anna Solini, Filip K. Knop, Tina Vilsbøll

**Affiliations:** ^1^Clinical Metabolic Physiology, Steno Diabetes Center Copenhagen, Gentofte Hospital, University of Copenhagen, Copenhagen, Denmark; ^2^Department of Clinical and Experimental Medicine, University of Pisa, Pisa, Italy; ^3^Department of Surgical, Medical, Molecular and Critical Area Pathology, University of Pisa, Pisa, Italy; ^4^Department of Clinical Medicine, Faculty of Health and Medical Sciences, University of Copenhagen, Copenhagen, Denmark; ^5^Novo Nordisk Foundation Center for Basic Metabolic Research, Faculty of Health and Medical Sciences, University of Copenhagen, Copenhagen, Denmark

**Keywords:** glucagon-like peptide-1, glucagon-like peptide-1 receptor agonist, glucagon, non-alcoholic fatty liver disease, non-alcoholic steatohepatitis

## Abstract

Along the obesity pandemic, the prevalence of non-alcoholic fatty liver disease (NAFLD), often regarded as the hepatic manifestation of the metabolic syndrome, increases worldwide representing now the prevalent liver disease in western countries. No pharmacotherapy is approved for the treatment of NAFLD and, currently, the cornerstone treatment is lifestyle modifications focusing on bodyweight loss, notoriously difficult to obtain and even more difficult to maintain. Thus, novel therapeutic approaches are highly demanded. Glucagon-like peptide-1 (GLP-1) receptor agonists (GLP-1RAs) are approved for the treatment of type 2 diabetes and obesity. They exert their body weight-lowering effect by reducing satiety and food intake. GLP-1RAs have also been shown to reduce liver inflammation and fibrosis. Furthermore, glucagon receptor agonism is being investigated for the treatment of NAFLD due to its appetite and food intake-reducing effects, as well as its ability to increase lipid oxidation and thermogenesis. Recent studies suggest that glucagon receptor signaling is disrupted in NAFLD, indicating that supra-physiological glucagon receptor agonism might represent a new NAFLD treatment target. The present review provides (1) an overview in the pathophysiology of NAFLD, including the potential involvement of GLP-1 and glucagon, (2) an introduction to the currently available GLP-1RAs and (3) outlines the potential of emerging GLP-1RAs and GLP-1/glucagon receptor co-agonists in the treatment of NAFLD.

## Introduction

Non-alcoholic fatty liver disease (NAFLD) is defined as fat accumulation in more than 5% of the hepatocytes. NAFLD can be subdivided according to the level of inflammation ranging from simple steatosis without inflammation to non-alcoholic steatohepatitis (NASH), which is often associated with fibrosis and over time may lead to cirrhosis and end-stage liver failure. NAFLD also increases the risk of hepatocellular carcinoma (HCC) (1).

The prevalence of NAFLD is increasing. Nearly 25% of the population in western countries have NAFLD and up to 6.5% fulfill the criteria of NASH (2). The increased prevalence of end-stage liver failure and HCC due to progressive NAFLD has led NAFLD to become the second most common indication for liver transplantation, likely configuring the leading indication for liver transplantation within the next two decades (3).

NAFLD is considered the “hepatic manifestation” of metabolic syndrome (4). Indeed, NAFLD is associated with central visceral adiposity except for a small proportion of lean patients, in whom genetic predisposition might play a crucial role in liver steatosis and fibrosis. Most morbidly obese patients undergoing bariatric surgery have NAFLD, nearly 30% have NASH, and 10% have advanced liver fibrosis (5). NAFLD is also closely linked with type 2 diabetes (T2D). In T2D the prevalence of NAFLD raises up to 70–75%, and the prevalence of NASH and advanced fibrosis are 65 and 15%, respectively (6). Importantly, coexisting T2D almost doubles the rate of which NAFLD progresses to end-stage liver disease and HCC, respectively (7, 8). A recent meta-analysis comprising nearly 300,000 individuals showed that patients with NAFLD have an increased risk of developing T2D compared to controls [hazard ratio (HR) 2.22, 95% CI 1.84–2.60], and that risk of T2D increases across the stages of NAFLD (9). In addition to T2D, NAFLD is accompanied and complicated by several other extra-hepatic manifestations. By stimulating pro-inflammatory and pro-thrombotic factors, it contributes to the development of several chronic diseases, including ischemic heart disease, cardiomyopathy, cardiac arrhythmias and chronic kidney disease. Noteworthy, the leading cause of mortality in NAFLD is cardiovascular disease (CVD) (10, 11).

No pharmacological therapies are approved for the treatment of NAFLD and lifestyle changes focusing on caloric restriction and weight loss constitute the general treatment recommendations. Recent trials investigating glucagon-like peptide-1 (GLP-1) receptor (GLP-1R) agonists (GLP-1RAs) for the treatment of NAFLD have shown promising results. Furthermore, GLP-1R/glucagon receptor dual agonists are being investigated for the treatment of NAFLD (12). In addition to its effects on glucose metabolism, glucagon is suggested to induce body weight loss, by increasing satiety and enhancing hepatic lipid oxidation and whole-body energy expenditure (13). This review provides (1) insights into the pathogenesis of NAFLD including the potential involvement of GLP-1 and glucagon, (2) a critical appraisal of the applicability of GLP-1RAs in NAFLD treatment, and (3) a review of the evidence for GLP-1/glucagon receptor co-agonism as a novel approach in the treatment of NAFLD.

## The pathogenesis of non-alcoholic fatty liver disease

**Figure 1:**
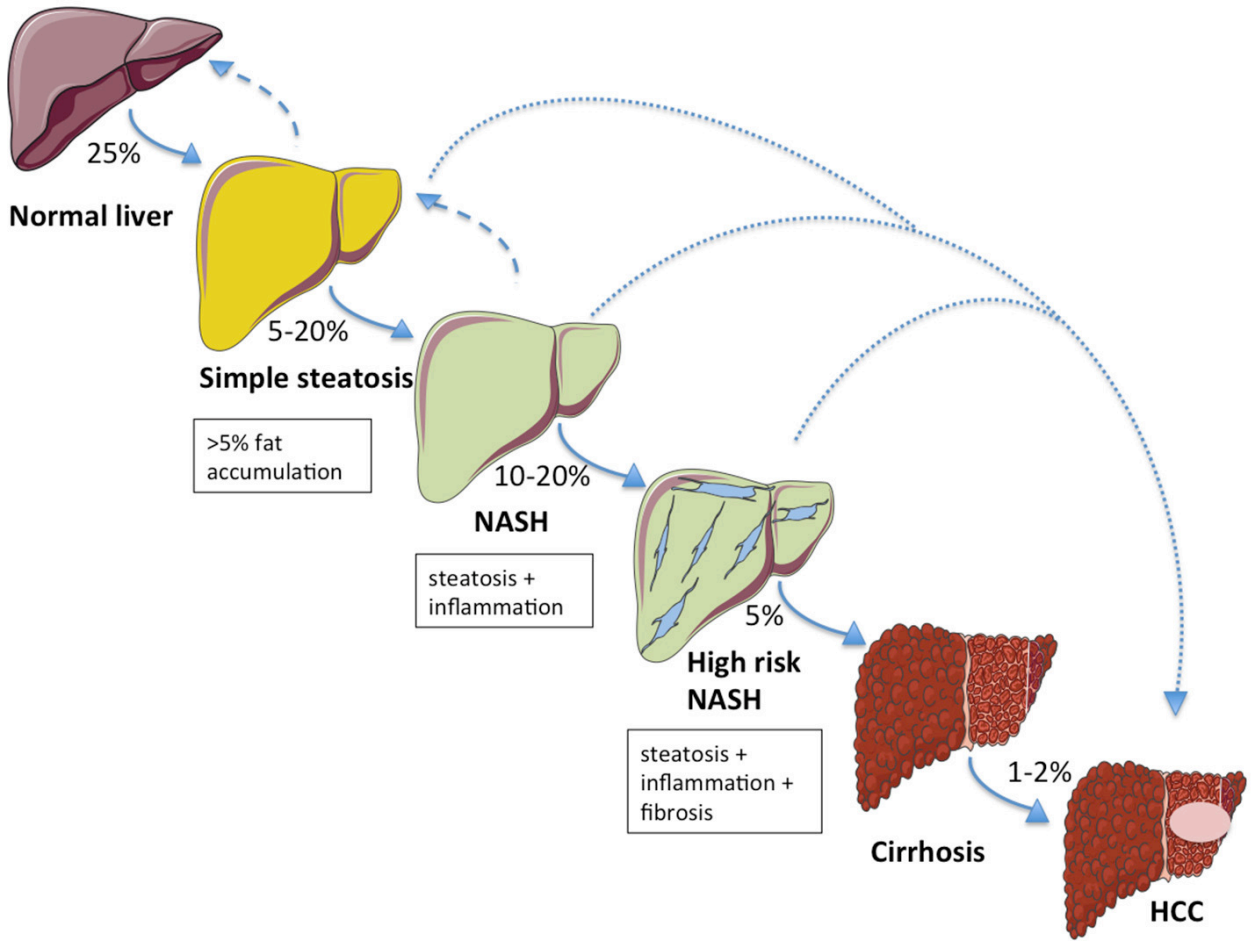
The NAFLD spectrum and relative probabilities to progress across the stages of liver damage. Aside to a classical development in the natural history of the disease, alternative routes (dotted lines) directly leading to hepatocellular carcinoma (HCC) from simple steatosis or NASH are possible. Hepatic steatosis and NASH are both reversible conditions (dashed arrows).

The first phase of NAFLD is characterized by accumulation of fat in the liver (hepatic steatosis), which may progressively lead to NASH (in 5–20% of patients) with or without concomitant fibrosis. Among the patients who develop NASH, 10–20% will progress to higher-grade fibrosis and approximately 5% will develop overt cirrhosis (14). It is arguable whether advanced fibrosis may regress, whereas steatosis and NASH are both reversible conditions (15). NAFLD-associated cirrhosis has traditionally been regarded as the leading risk factor for the development of HCC. However, HCC may also occur in a non-cirrhotic liver (16, 17). This suggests that NAFLD might not necessarily implicate a sequential process to evolve (Figure 1) (18).

**Figure 2:**
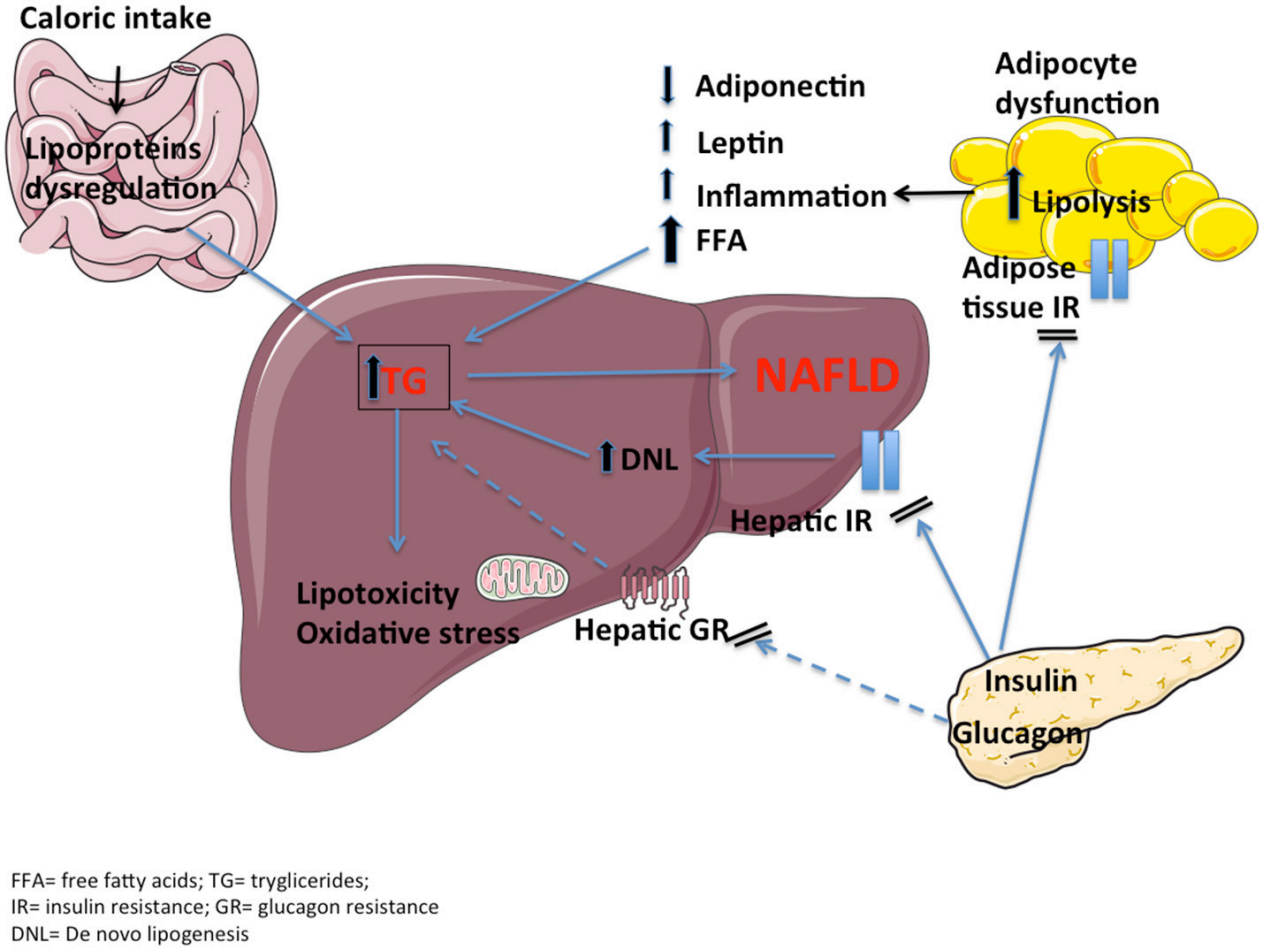
The pathophysiology of NAFLD includes dietary fat contribution, hepatic and adipose tissue insulin resistance, proinflammatory cytokines, lipotoxicity and oxidative stress. A reduced hepatic glucagon resistance (dashed lines), together with an impaired incretin effect, may be additional mechanisms.

Triglycerides (TG) accumulation is likely one of the first steps in the pathophysiology of NAFLD as a result of an impaired free fatty acid (FFA) metabolism in the liver (Figure 2). Excessive caloric intake increases FFA load to the liver to a point that the ability of the hepatocytes to oxidize FFA or re-esterify to TG and secrete very low-density lipoproteins (VLDL) is overwhelmed. Thus, TG accumulate in forms of lipid drops (steatosis). Moreover, insulin resistance of the adipose tissue, associated with overweight/obesity, contributes to the flux of FFA from adipose tissue to the liver through unrestricted lipolysis (19). Lastly, increased *de novo* lipogenesis, i.e., hepatic FFA synthesis, seems to contribute to lipid deposition (20). Prolonged accumulation of lipids in hepatocytes is associated with lipotoxicity, which may initiate inflammation, apoptosis and ultimately fibrosis (21). The main route of hepatic fat oxidation is the mitochondrial tricarboxylic acid (TCA) cycle. An overactive TCA cycle stresses the endoplasmic reticulum, thus inducing mitochondrial dysfunction and formation of reactive oxidative species and toxic lipid intermediates, like ceramides and diacylglycerol (22, 23). Insulin resistant adipose tissue may also enhance inflammation by lowering release of anti-inflammatory adipokines such as adiponectin and increasing release of leptin and pro-inflammatory cytokines like interleukin 6 (IL-6) and tumor necrosis factor-alpha (TNF-α) (24). This inflammatory milieu may contribute to hepatic insulin resistance and thus establish a vicious circle. At a molecular level, serine phosphorylation of insulin receptor substrate-1 (IRS-1) by inflammatory signals appears to be one of the key aspects that disrupt insulin-receptor signaling (25).

The gut-derived incretin hormones GLP-1 and glucose-dependent insulinotropic polypeptide (GIP) are responsible for the so-called incretin effect (i.e., the potentiation of glucose-stimulated insulin secretion after meal ingestion) (26). Additionally, GLP-1 suppresses glucagon release from pancreatic alpha cells, delays gastric emptying and enhances satiety (27). While GIP displays similar insulinotropic properties, it has been shown to act as a bifunctional blood glucose stabilizer by stimulating glucagon release in the presence of low plasma glucose levels. Moreover, GIP receptor activation has reported contrasting effects on satiety, caloric intake and body weight (28). It has been suggested that patients with NAFLD have lower concentrations of biologically active incretin hormones compared to healthy individuals, which may be a consequence of an increased degradation by dipeptidyl peptidase 4 (DPP-4) (the enzyme, which under normal conditions inactivates the incretin hormones) (29) or a decreased production (30, 31). Conversely, a series of studies by our group suggest that patients with NAFLD have normal GLP-1 and GIP plasma levels, even though they exhibit a reduced incretin effect (32). Whether a reduced incretin effect (reduced beta cell sensitivity to GIP and/or GLP-1) may play a role in the pathophysiology of NAFLD warrants further investigations.

Glucagon is a key hormone in the regulation of overall energy homeostasis during the fasting state and other energy-demanding situations. Beyond the stimulation of hepatic glucose production, it also affects hepatic fat metabolism promoting lipid oxidation and lowering lipid synthesis. Glucagon decreases food intake and appetite by central mechanism and by reducing gastric emptying (33, 34). Furthermore, glucagon may display thermogenic properties, inducing an increase in energy expenditure through brown adipose tissue activation (13, 35). It has been hypothesized that hepatic glucagon resistance might play an important role in fat accumulation in the liver and *vice versa* (36). Preclinical studies in NAFLD have demonstrated that a reduction in G protein-coupled glucagon receptor (GCGR) signaling results in an increase of hepatic fat content (37, 38). Moreover, a recent study from *Guzman* and colleagues (39) has shown that, in patients with T2D, treatment with a selective GCGR antagonist, LY2409021, induces a significant increase in hepatic lipid content assessed by magnetic resonance imaging, suggesting that GCGR activation is required to prevent build-up of fat in the hepatocytes. It has been hypothesized that a reduction in hepatic GCGR and signaling molecules affects a feedback mechanism acting on the pancreatic alpha cells, increasing glucagon secretion, and this liver-pancreas axis might contribute to fasting hyperglucagonemia (36). In line with this hypothesis, results from our group show that individuals with NAFLD (both normoglycemic individuals and patients with T2D) exhibit significantly higher fasting plasma glucagon levels compared to matched controls without NAFLD (28). However, whether hyperglucagonemia is directly involved in the pathogenesis of NAFLD or is a consequence of steatosis remains uncertain.

## Treatment of NAFLD: applicability of GLP-1RAs

### Potential modes of action of GLP-1RAs in NAFLD

**Figure 3:**
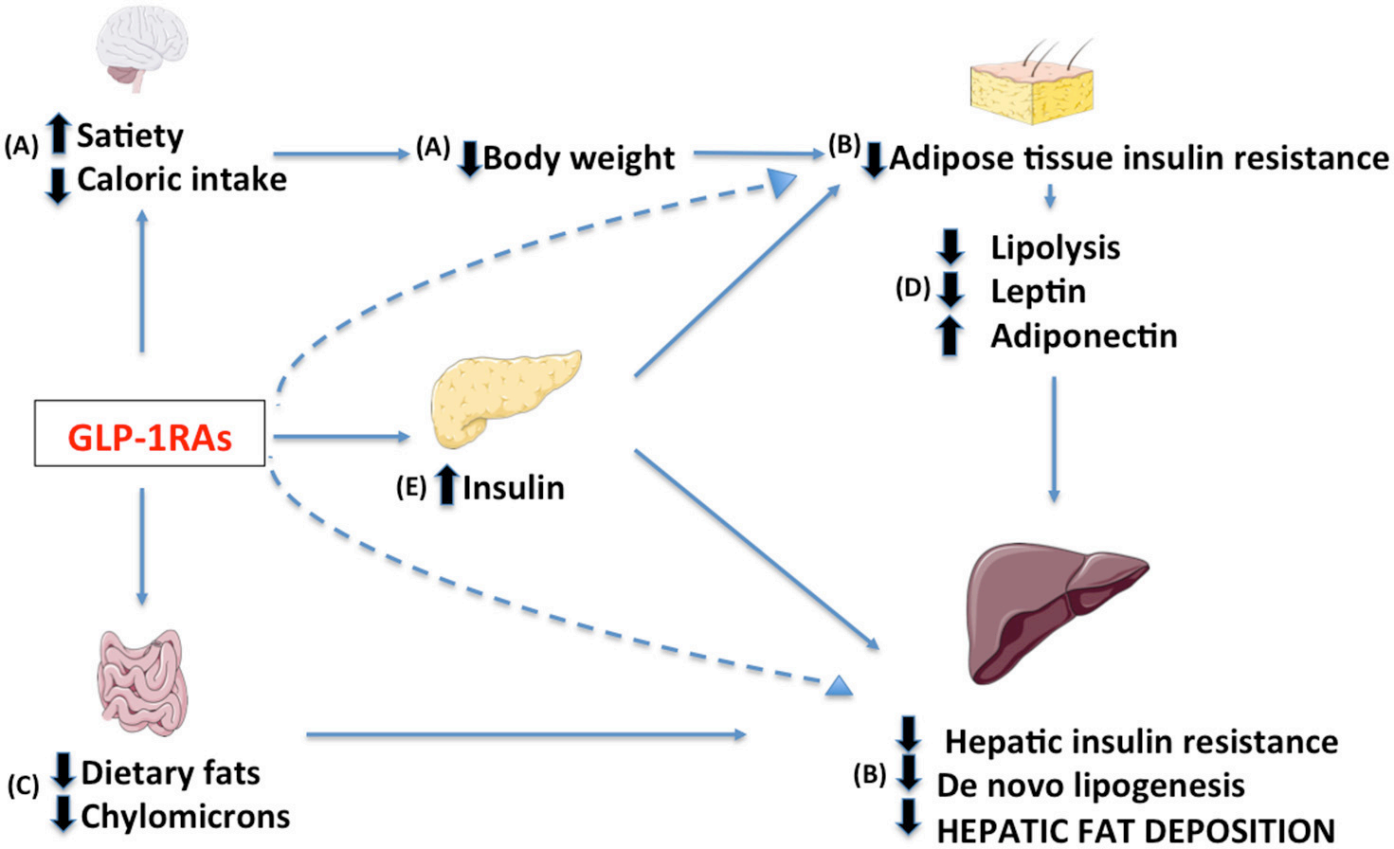
Potential targets of GLP-1 receptor agonists in the treatment of NAFLD include: **(A)** a decrease in caloric intake through central regulation of satiety, **(B)** a reduction of hepatic and adipose tissue insulin resistance due to decrease in body weight, (a direct effect may not be excluded, dashed lines) **(C)** a modified intestinal lipoprotein metabolism, **(D)** a resolution of dysfunctional adipose tissue and **(E)** an enhancement of insulin release.

Current NAFLD treatment consists of interventions promoting bodyweight loss. It has been estimated by studies with ^1^H-magnetic resonance spectroscopy that decreasing bodyweight by 10% via diet combined with physical activity can induce a reduction in hepatic TG concentration up to nearly 60% in overweight individuals (40). Bariatric surgery is the most effective treatment in severely obese patients, inducing significant improvement in lobular inflammation and a disappearance of NASH in 50–85% of cases (41). Currently no pharmacological treatment has proven efficacious, however numerous drugs targeting key-steps in NAFLD pathogenesis are under investigation. These compounds can be grouped in medications targeting (1) metabolic derangements including excess bodyweight, (2) inflammation and oxidative stress, and (3) dysregulation of the gut-liver axis (42). In this regard GLP-1RAs exhibit potent metabolic effects, however they might also affect other of the proposed targets. In the following paragraphs, we will present the potential mechanisms of action of GLP-1RAs in NAFLD provided by studies in humans (Figure 3).

#### Metabolic effects

##### Bodyweight reduction

GLP-1 has a documented dose-dependent effect on satiety, through central mechanisms in the hypothalamus and brainstem. Accordingly, a reduced caloric intake has been observed in lean and obese individuals and in patients with T2D after exogenously administered GLP-1 during *ad libitum* meals. In addition, weight loss is a consistent finding in clinical trials investigating GLP-1RAs (27).

##### Reduction of hepatic and adipose tissue insulin resistance

An improved insulin sensitivity is expected after chronic treatment with GLP-RAs mainly due to marked bodyweight reductions. However, this effect might be independent of changes in visceral fat accumulation since previous studies have demonstrated that the hepatic glucose production is decreased in healthy individuals following acute administration of both native GLP-1 (43) and the GLP-1RA exenatide (44). Furthermore, in patients with NASH, GLP-1 decreases *de novo* lipogenesis and reduces levels of lipolysis-induced FFA and triglyceride-derived toxic metabolites (45). Whether these actions could be partly mediated through hepatic GLP-1R signaling remains uncertain, as the presence of GLP-1Rs in the liver has not been confirmed (46–48).

##### Insulinotropic effect

As alluded above, patients with NAFLD, in whole its spectrum including cirrhotic individuals, show a reduced incretin effect. Whether the insulinotropic action of GLP-1RAs, overcoming the reduced incretin effect, might ameliorate NAFLD is however still uncertain.

#### Inflammation and oxidative stress

The impact of GLP-1RAs on hepatic lipotoxicity has been extensively explored in cellular and animal models, whereas few clinical studies have been conducted (49). In NAFLD patients, the increase in serum concentrations of total adiponectin following GLP-1RAs treatment may be consistent with a restoration of a dysfunctional adipose tissue (50). Liraglutide also decreases fasting serum leptin resulting in a significant reduction in the leptin-to-adiponectin ratio (45). In turn, adiponectin can ameliorate NAFLD-associated liver abnormalities by regulating the oxidation of hepatic fatty acid and the activity of acetyl-CoA carboxylase and fatty acid synthase, two key enzymes involved in fatty acid synthesis (51).

#### Gut-liver axis

Lipoproteins production by the liver and by the intestine is subject to a variety of hormonal and nutritional modulators and is deranged in T2D as well as in insulin resistant states including NAFLD (52). As carefully reviewed by Xiao et al. numerous studies have demonstrated that GLP-1RAs may ameliorate postprandial lipidemia during meal tests by multiple pathways including decreased absorption of dietary fats as consequence of reduced gut motility and direct inhibition of chylomicron synthesis and secretion (53).

### GLP-1RAs and clinical trials in NAFLD

#### Lixisenatide

In a systematic meta-analysis including 12 randomized controlled trials (RCTs) comparing lixisenatide to placebo or active interventions in T2D, lixisenatide was reported to normalize levels of alanine aminotransferase (ALT) in a greater proportion of overweight and obese patients with T2D than comparators (54). However, at present, no trial aimed at testing the efficacy of lixisenatide in patients with NAFLD has been conducted.

#### Exenatide

Almost all human studies with exenatide twice-daily evaluating NAFLD-related endpoints involve patients with T2D. Several case series (55, 56) and open-label trials (57–60) suggest that the combination of a better glycemic control, improved metabolic parameters and bodyweight reductions achieved by exenatide treatment as monotherapy or as add-on to standard therapies may lead to improvements in liver biomarkers and hepatic fat reductions in patients with T2D. However, whether exenatide is able to ameliorate histological features of NAFLD/NASH has not been investigated by RCTs.

#### Liraglutide

Liraglutide is the only GLP-1RA, which has been investigated for the treatment of NAFLD. In the “Liraglutide Efficacy and Action in Diabetes” (LEAD) programme (61), a total of 154 patients with T2D within the LEAD-2 trial participated in a sub-study to assess liver fat content by the liver-to-spleen attenuation ratio at a computer tomography (CT) scan. Such ratio significantly increased from baseline after 26 weeks of treatment with liraglutide 1.8 mg/day, indicating a reduction in liver steatosis, whereas it was unchanged in patients treated with lower doses of liraglutide, glimepiride or placebo. Liraglutide was also associated with a reduction in mean ALT levels, which, however, disappeared after correction for changes in weight and HbA_1c_ (62, 63). An open-label uncontrolled trial including 27 patients with T2D and NAFLD treated with liraglutide 0.9 mg/day for 24 weeks showed a trend toward increases in liver-to-spleen attenuation ratio assessed by CT scan and, more importantly, a significant improvement in histological inflammatory scores in 10 subjects undergoing liver biopsies after a prolonged treatment of 96 weeks (64). In patients with poorly controlled T2D, 6 months of treatment with liraglutide 1.2 mg/day significantly reduced liver fat content as evaluated by ^1^H-magnetic resonance spectroscopy, an effect mainly driven by bodyweight loss (65). The first RCT to investigate the effect of liraglutide in patients with NAFLD was the LEAN study, in which 52 patients with NASH were randomized to either 48 weeks of treatment with liraglutide (1.8 mg/day) or placebo (66). In this study, 39% of the patients treated with liraglutide achieved histologic resolution of NASH as compared to 9% in the control group (*p* = 0.019). Furthermore, worsening of fibrosis was significantly reduced with liraglutide compared to placebo. With respect to the previously mentioned study, this proof-of concept study has important methodological strengths residing in the availability of baseline and post-treatment hepatic biopsies. Interestingly, reductions in bodyweight and HbA1_c_ were similar in patients with improvements in liver histology (“responders”) and those without (“non-responders”), suggesting that, beyond bodyweight reduction and improvement in glucose control, other mechanisms may be involved in the beneficial effects of liraglutide. The effect of liraglutide in NAFLD has also been compared to other antidiabetic drugs. In 87 patients with NAFLD and T2D, 12-week treatment with liraglutide 1.8 mg/d resulted in similar reductions in intra-hepatic fat as treatment with metformin, whereas liraglutide was significantly more effective than gliclazide. In this trial, no differences in bodyweight changes or glucose control among treatments were observed (67). In one study, 12-week treatment with insulin glargine *vs* liraglutide (mean dose = 1.3 mg/day) showed similar glycemic control and decrease of hepatic fat burden, although a reduction in BMI was only observed in the liraglutide-treated patients (68). Furthermore, in a 12-week RCT in which patients were either assigned to liraglutide (1.8 mg/day), sitagliptin (100 mg/day) or placebo, no difference in glycemic control or bodyweight reductions were found, and both treatments failed to reduce liver hepatic fat content compared to placebo (69). A recent randomized study comparing structured lifestyle modification (a cornerstone in the currently recommended treatment of NASH) to liraglutide 3 mg/day without lifestyle modifications showed similar reductions in ALT and liver stiffness with no difference in bodyweight variations. This findings indicate that an additive effect of lifestyle modifications and liraglutide 3 mg/day might exist (70).

#### Semaglutide

An ongoing phase 2 trial (NCT02970942) is currently investigating the efficacy and safety of three doses of subcutaneous semaglutide once-daily versus placebo in subjects with NASH. It will include 288 participants. As in the LEAN study, the primary outcome is histologic resolution of NASH without worsening of fibrosis after 72 weeks of treatment, while secondary outcomes include improvement in liver fibrosis (≥1 stage) with no worsening of NASH, NAFLD activity score, as well as multiple serum markers of fibrosis. Furthermore, a RCT (NCT03357380) is currently comparing the change in early stages of scar tissue as well as fat deposition in the liver, as detected by magnetic resonance imaging scans, in patients treated with semaglutide or placebo for 72 weeks.

An overview on the clinical trials investigating GLP-1RAs treatment in NAFLD is presented in Table 1.

**Table 1:** Clinical trials with GLP-1RAs in NAFLD.

**Subjects (number)**	**Study type**	**Diagnostic test**	**Intervention (dose)**	**Comparator (dose)**	**Duration (weeks)**	**Outcome evaluation**	**Effect**	**References**
T2D (8)	Case series	Biopsy-proven NAFLD/NASH	Exe (10 μgx2/d)	**–**	28	Liver biopsy	**(**+**)** histological inflammation and fibrosis	(55)
T2D overweight/obese (974)	Single arm-trial	Liver enzymes	Exe (10 μgx2/d)	**–**	104	Liver enzymes	+ 39% of subjects with elevated baseline ALT had normalization of ALT	(57)
T2D obese (125)	Open-label	FLI	Exe (10 μx2/d alone or add-on Met and/or SU)	Met and/or SU	26	FLI	+ improvement of FLI in Exe group vs. oral antidiabetic agents	(58)
T2D overweight/obese (60)	RCT	Liver enzymes; Ultrasound	Exe (10 μgx2/d)	Intensive treatment with insulin glargine	12	Liver enzymes; Ultrasound	+ reduction of liver enzymes and degree of fatty liver at ultrasound with Exe vs. Glargine	(59)
T2D overweight (117)	RCT	Liver enzymes	Exe (10 μgx2/d)	Met	12	Liver enzymes	+ reduction of liver enzymes	(60)
T2D overweight/obese (27)	Single arm-trial	Biopsy-proven NAFLD/NASH	Lira (0.9 mg/d)	–	24 96 (*N* = 10)	CT scanLiver biopsy (*N* = 10)	**(**+**)** liver to spleen attenuation ratio + histological inflammation and fibrosis	(64)
T2D overweight/obese (68)	Single arm-trial	^1^H-MR spectroscopy	Lira (1.2 mg/d)	–	26	^1^H-MR spectroscopy	+ 31% liver fat content	(65)
Overweight/obese (17 T2D out of 52)	RCT	Biopsy-proven NASH	Lira (1.8 mg/d)	Placebo	48	Liver biopsy	++ 39% histological resolution of NASH with Lira vs. 9% with Placebo	(66)
T2D (154)	RCT (LEAD-2 substudy)	CT scan	Lira (1.8-1.2-0.6 mg/d) add on- Met	Glimepiride (4 mg) or Placebo add on- Met	26	CT scan	+ 10% liver to spleen attenuation ratio with Lira 1.8 mg/day vs. no effect with other treatments	(62)
T2D (87)	RCT	Ultrasound	Lira (1.8 mg/d)	Met or Gliclazide	24	Ultrasound	+ improvement in hepatic/renal ratio index in Lira vs. Gliclazide	(67)
T2D overweight/obese (35)	RCT	^1^H-MR spectroscopy; MR imaging	Lira (0.6 to 1.8 mg/d)	Insulin glargine titrated to achieve FPG < 7mM	12	^1^H-MR spectroscopy and MR imaging	±no difference in liver fat reduction between Lira and Glargine	(68)
T2D overweight/obese (52)	RCT	^1^H-MR spectroscopy	Lira (1.8 mg/d)	Sitagliptin (100 mg)Placebo	12	^1^H-MR spectroscopy	± no effect in liver fat content with Lira, as well as Sitagliptin or Placebo	(69)
NonT2D obese	RCT	MR imaging	Lira (3 mg/d)	Intensive lifestyle intervention	26	MR imaging	± no difference in liver fat reduction between Lira and lifestyle intervention	(70)
288 patients with T2D (288)	RCT	Biopsy-proven NASH	Sema (0.1-0.2-0.4 mg/d)	Placebo	72	Liver biopsy	Ongoing (NCT02970942)	
T2D (66)	RCT	MR imaging	Sema (0.4 mg/d)	Placebo	72	MR imaging	Ongoing (NCT03357380)	

*d, day; FLI, fatty liver index; FPG, fasting plasma glucose; Lira, liraglutide; MR, Magnetic resonance; Met, metformin; NAFLD, Non-alcoholic fatty liver disease; NASH, Non-alcoholic steatohepatitis; Sema, semaglutide; SU, sulphonylurea; T2D, type 2 diabetes; (+), trend improvement in predefined outcome; +, significant improvement in predefined outcome; ++, significant improvement in predefined outcome judged by gold-standard method; ±, no significant effect in predefined outcome*.

## Emerging GLP-1 and glucagon receptor co-agonists

Multiple GLP-1R co-agonists are emerging for the treatment of obesity and diabetes. These agents, comprising GLP-1 combined molecules with glucagon or other hormones, have been investigated in preclinical studies for NAFLD treatment. Combination therapy and hybrid molecules that act through multiple receptors appear to maximize the beneficial outcomes, without increasing side effects of the single molecules. GLP-1 and glucagon display similar amino acid N-terminal sequences and bind to structurally related receptors, facilitating the development of single-molecule GLP-1R/GCGR co-agonists. In general, GLP-1 and glucagon are believed to antagonize their respective effects on glucose homeostasis. Whereas GLP-1 decreases plasma glucose levels by exerting insulinotropic effects, glucagon stimulates hyperglycemia by enhancing hepatic glucose output. However, novel dual receptor agonists have been developed for the treatment of obesity and T2D under the concept that GLP-1 restrains the hyperglycemic action of glucagon, while allowing it to exert its beneficial actions on bodyweight, food intake, lipid metabolism and thermogenesis (71).

One month-therapy with a pegylated GLP-1R/GCGR dual agonists in diet-induced obese (DIO) mice resulted in bodyweight loss and improved glycemic control. These effects were coupled to an amelioration in lipid metabolism and hepatic steatosis, which markedly exceeded the effect of single GLP-1RA treatment (72). In 2016, another balanced dual receptor agonist demonstrated pronounced effect on bodyweight and glucose control, together with reducing hepatic fat content in rodents and non-human primates (73). Recently, chronic exposure of a GLP-1/glucagon dual analog conjugated with maleimide showed beneficial effects on liver morphology in DIO mice (74).

Oxyntomodulin, a gut-derived peptide hormone, activates both the GLP-1R and the GCGR, although, with reduced affinity compared to GLP-1 and glucagon, respectively. Oxyntomodulin has already shown to reduce food intake and bodyweight in rodents and humans (75, 76). Interestingly, in a mouse model of NASH, 2 weeks of treatment with a oxyntomodulin analog also ameliorated the hepatic histopathological features of this disease (77).

In addition to GLP-1R/GCGR dual agonists, a chimeric peptide as dual GLP-1/GIP receptor agonists has been developed, showing enhanced therapeutic potential for obesity and related comorbidities. When compared to single agonists, unimolecular dual incretin was more effective in correcting adiposity-induced insulin resistance in animal models of obesity and diabetes; it also improved liver function by reversing hepatic steatosis features in histopathological specimens of DIO mice. In healthy and diabetic subjects, the co-agonist displayed to improve glucose tolerance and insulin secretion, although no data regarding bodyweight, lipid metabolism, or liver function were reported (78). However, preliminary studies in non-diabetic obese individuals showed that simultaneous activation of GLP-1 and GIP receptors did not potentiate GLP-1-mediated effects in lowering food intake and appetite (79).

This approach with dual agonists has been followed by the development of monomeric triagonists, incorporating residues from GLP-1, glucagon and GIP. In high-fat diet fed mice, treatment with the triagonist dose-dependently improved steatohepatitis and reduced levels of ALT, underpinning the potential for this compound to treat liver disease (80). While multi-agonism with incretin hormones and glucagon has demonstrated great therapeutic potential, the conceptual approach of polypharmacology has also been extended to other hormone combinations. In pre-clinical studies, GLP-1 mediated delivery of estrogen or dexamethasone has proven beneficial effects on glucose tolerance, bodyweight control and systemic inflammation (81). Interestingly, a conjugated glucagon and thyroid hormone (T_3_) agonist has shown to reverse metabolic syndrome related abnormalities (82). In a rodent model of NASH, 3-week treatment with glucagon/T_3_ lowered ALT levels and improved macroscopic and histological features of NASH, including reversal of fibrosis (82).

## Conclusion and future perspectives

New knowledge about the pathophysiology of NAFLD has been accumulating over the last decade, displaying the complexity of the mechanisms involved in the development of this condition. In addition to bodyweight loss through lifestyle interventions, pharmacotherapies targeting adipose tissue, the digestive system (gut-liver axis) and/or inflammation are warranted. In this perspective, GLP-1RAs may act through all of these different pathways. However, most of the available GLP-1RAs have still not been thoroughly investigated for the indication of NAFLD. Liraglutide treatment has been shown to improve NASH histology and reduce progression of fibrosis (66). Clinical trials investigating the newly approved GLP-1RA semaglutide for the treatment of NASH are currently ongoing. Another approach, which seems promising for future treatment of NAFLD, is the combination of GLP-1 and glucagon, since the latter may potentiate incretin-mediated weight loss and increase lipid utilization and FFA oxidation in the liver. With the additional development of multiple new dual- and tri-agonist, GLP-1 and glucagon-based poly-agonists in the treatment of NAFLD represent an exciting novel pharmacological approach. Whether the promising preclinical pharmacology will result in successful clinical trials is a question that will be answered in coming years.

## Authors contributions

MS researched the data, made substantial contributions to the discussion of the content, wrote the first draft and reviewed/edited the manuscript. AC, AA, AS, FK, and TV made substantial contributions to the discussion of the content and reviewed/edited the manuscript.

### Conflict of interest statement

Within the last 36 months, FK has served on scientific advisory panels and/or been part of speaker's bureaus for, served as a consultant to and/or received research support from Amgen, AstraZeneca, Boehringer Ingelheim, Eli Lilly, Gubra, MedImmune, MSD/Merck, Mundipharma, Norgine, Novo Nordisk, Sanofi and Zealand Pharma; FK is a minority shareholder in Antag. The remaining authors declare that the research was conducted in the absence of any commercial or financial relationships that could be construed as a potential conflict of interest
